# Natural History and Treatment Strategies of Advanced PEComas: A Systematic Review

**DOI:** 10.3390/cancers13205227

**Published:** 2021-10-18

**Authors:** Agathe Bourgmayer, Simon Nannini, Paul Bonjean, Jean-Emmanuel Kurtz, Gabriel G. Malouf, Justine Gantzer

**Affiliations:** 1 Department of Medical Oncology, Strasbourg-Europe Cancer Institute (ICANS), 67033 Strasbourg, France; agathe.bourgmayer@gmail.com (A.B.); s.nannini@icans.eu (S.N.); je.kurtz@icans.eu (J.-E.K.); g.malouf@icans.eu (G.G.M.); 2 Department of Clinical Research and Pharmacology Innovation Unit, CHU de Saint-Etienne, Hôpital Nord, 42270 Saint-Priest en Jarez, France; paul.bonjean@orange.fr; 3 Davidson Team, Department of Cancer and Functional Genomics, INSERM UMR_S1258, Institute of Genetics and Molecular and Cellular Biology, 67400 Illkirch, France

**Keywords:** PEComa, locally advanced, metastatic, outcomes, risk factors

## Abstract

**Simple Summary:**

In this article, we review the clinical features of advanced PEComas and show the diversity of reported data among authors, emphasizing the heterogeneity of molecular characterization and treatment strategy. Based on clinical data collected from 124 case reports, metastatic disease at diagnosis and a grouped version of the Bleeker’s risk category were the only factors significantly associated with death. Due to a significant number of missing data or short follow-ups, results regarding prognostic factors should, however, be interpreted with caution.

**Abstract:**

PEComas is a family of rare mesenchymal tumors. This systematic review aims to better understand the natural history of advanced PEComas. After a search on the PubMed database and main oncology meeting libraries according to the PRISMA guidelines, 88 articles reported in the English literature were included. Data on clinical and histological features, treatments and outcomes were collected. To identify risk factors, univariate and multivariate analyses were performed. Seven cohorts of patients and 124 individual patients were identified. Focusing on case reports, most patients were metastatic, and the median overall survival (OS) of the entire cohort was 60 months (95%CI 33; NA). Risk factors significantly associated with OS in the multivariate analysis were the presence of metastasis at diagnosis (HR: 2.59, 95%CI 1.06; 6.33, *p* = 0.036) and the grouped-Bleeker’s risk category (HR: 4.66; 95%CI 1.07; 20.19; *p* = 0.039). In the metastatic population, only the presence of lymph node metastasis was associated with OS (HR: 3.11; 95%CI 1.13; 8.60, *p* < 0.05). Due to a lack of events, it was not possible to conclude on other factors. This review of the literature highlights the heterogeneity of literature data and shows the great diversity of clinical management strategies.

## 1. Introduction

Perivascular epithelioid cell neoplasm or PEComa is a family of rare mesenchymal tumors composed of “perivascular epithelioid cells” or “PEC” with melanocyte and smooth muscle differentiation. Since their first descriptions in the early 1990s, mostly in lung and kidney locations, tumors sharing these common features were described in a variety of anatomic locations [1,2,3,4,5]. The 2020 World Health Organization (WHO) latest classification describes PEComas as “mesenchymal tumors composed of histologically and immunohistochemically distinctive perivascular epithelioid cells” [6,7,8]. 

Currently, this family includes angiomyolipoma (AML), lymphangioleiomyomatosis (LAM), clear cell “sugar” tumor of the lung (CCST), clear cell myomelanocytic tumor of the falciform ligament/ligamentum teres (CCMT) and other PEComas known as PEComa-not otherwise specified (NOS) of visceral, bone and soft tissue sites [9]. Regarding molecular characteristics, PEComas are characterized by mutations leading to mTOR pathway activation, such as *TSC1* or *TSC2* bi-allelic inactivation, *TFE3* gene fusion and *FLCN* truncating mutations [10]. This activation was first discovered exploring the high risk for patients with tuberous sclerosis complex (TSC) disease to develop LAM or AML [11,12]. 

Some PEComas are indolent but others may have a malignant evolution with a poor prognosis. For example, the epithelioid AML, a variant of the classical form of AML, is a well-known aggressive tumor [13,14]. In 2005, Folpe et al. identified six tumor characteristics as poor prognostic factors in order to facilitate the assessment of the tumor’s aggressiveness [15].

Even today, the natural history of metastatic PEComa is still poorly understood due to its scarcity. Case reports and literature reviews have been previously published but often focused on a specific primary location, preventing conclusions to all PEComas. The last review article on a wide variety of primary PEComa-NOS was published in 2012 and proposed a more convenient, revised set of risk stratification criteria, only based on tumor size and mitotic index [16]. 

Since this last review, many articles, even though most of them were case reports, have been published. No clear recommendations are available on treatment strategies and, until very recently, data were lacking to discuss treatment sequences. Indeed, the latest published data (AMPECT trial, retrospective case series) argued to place mTOR inhibitors as the most efficient first line treatment based on the activation of the mTOR pathway [17,18].

In the present work, we aim to summarize the available data on locally advanced or metastatic PEComas-NOS, including tumor characteristics, treatments and outcomes in order to better understand this rare family of neoplasms and improve their management.

## 2. Materials and Methods

This review was conducted according to the Preferred Reporting Items for Systematic Reviews and Meta-Analyses (PRISMA) guidelines and registered in PROSPERO (registration number 279341) [19]. Three researchers took part in the review of all articles and discussed any discrepancies before data selection.

### 2.1. Literature Search

The systematic search was performed in June 2021, using different queries on the PubMed database and the three most relevant annual oncology meetings libraries.

To search in the PubMed database, we used the following sentences restricted to the title and/or in the abstract and to the species “human” as queries: (“PEComa” OR “Perivascular Epithelioid Cell Tumor”) AND (“metastatic” OR “metastasis” OR “locally advanced”); (“PEComa” OR “Perivascular Epithelioid Cell Tumor”) AND “systemic treatment”; (“PEComa” OR “Perivascular Epithelioid Cell Tumor”) AND (“mTOR inhibitor” OR “chemotherapy”). The Mesh Term “Perivascular Epithelioid Cell neoplasms’’ was not used for the final search as it was unexpectedly only mentioned in a minority of reports.

To search meeting abstracts, we used the keyword “PEComa” in the American Society of Clinical Oncology (ASCO), Connective Tissue Oncology Society (CTOS) and of European Society of Medical Oncology (ESMO) libraries. The references list of selected articles was further checked to identify additional case reports or series of interest that were missed by the previous queries.

### 2.2. Inclusion and Exclusion Criteria

All articles and abstracts were combined in a unique file including the title, year of publication and authors. By reading this information, we removed duplicated articles and abstracts originating from the same cohorts but presented at different timepoints in different meetings. From the PubMed search, we included case reports and retrospective case series. We defined “locally advanced tumor” as a non-metastatic tumor that cannot be only treated with localized treatment, such as surgery and/or radiotherapy, but requires systemic treatment alone or in combination with localized treatment. We excluded non-English articles, reviews and articles on localized disease, on other PEComas than the NOS or epithelioid AML subtypes or reporting only pathological and/or radiological data. From the meeting libraries search, we included only abstracts on case series with clinical data, without any published article since the meeting.

### 2.3. Data Extraction

Data collected from case reports were: age, gender, size of the primary tumor, Folpe criteria (tumor size greater than 5 cm; vascular invasion, mitotic index ≥1/50 high power field (HPF), high nuclear grade/cellular atypia, necrosis and infiltrative growth pattern), Folpe risk category, Bleeker adapted risk category, margins status (if surgery), site of the primary tumor, details on immunohistochemistry (IHC) markers (HMB45, MelanA, SMA, TFE3, PNL2 and Cathepsin K), details on mutational status, disease extension at diagnosis, site of metastatic lesions, treatment(s) received at the different timelines and outcomes.

Data collected from case series were: number of patients, median age, gender proportion, median size of the primary tumor, site of the primary tumor, disease extension at diagnosis, site of metastatic lesions, median follow-up, treatment(s) received as different lines and outcomes.

### 2.4. Statistical Analysis

Patient characteristics were described using numbers and proportions for categorical variables and using the mean, standard deviation, median and interquartile range for continuous variables.

To determine prognostic factors associated with progression free survival (PFS), a univariate analysis was performed on the following explanatory variables: age, tumor size (<5 vs. ≥5 cm), vascular invasion, mitotic index, grade, presence of tumor necrosis, infiltrative or non-infiltrative growth pattern, Folpe risk category, Bleeker risk category, TFE3 positive expression by IHC, surgery in the non-metastatic stage, chemotherapy in the non-metastatic stage, radiotherapy in the non-metastatic stage and mTOR inhibitor in the non-metastatic stage. 

In order to conclude on Bleeker’s risk category, it had to be redefined as two groups (benign/uncertain malignant potential PEComas and malignant PEComas) and named “Bleeker’s grouped-risk category”. The univariate analysis was performed using the univariate Cox model test and a Kaplan-Meier survival curve was performed for each variable. A multivariate Cox model was then built keeping age and other variables with a *p*-value <0.1 in univariate analysis, except for the mitotic rate, which was already included in Bleeker’s classification. Patients with missing data for at least one variable of interest were excluded from the multivariate model, and their characteristics were compared with those of retained patients to detect potential attrition bias.

Prognostic factors associated with the overall survival (OS) were also studied with a univariate Cox model for the following variables: age, primary tumor location, tumor size (<5 vs. ≥5 cm), vascular invasion, mitotic index, grade, presence of tumor necrosis, infiltrative or non-infiltrative growth pattern, Folpe risk category, Bleeker risk group, TFE3 positive expression by IHC, metastasis at diagnosis, type of treatment at diagnosis (surgery alone vs. surgery + adjuvant therapy) and type of systemic treatment at diagnosis (none vs. chemotherapy vs. mTOR inhibitors). The multivariate analysis was performed using the same criteria as in the PFS analysis. 

Similarly, prognostic factors associated with OS in the subgroup of metastatic patients were explored for the following variables: age, presence of metastasis at diagnosis, number of metastases (≤3 vs. >3), presence of lung metastasis, presence of lymph node metastasis, presence of liver metastasis, treatment strategies: surgery, radiotherapy and chemotherapy or mTOR inhibitors. The multivariate Cox model was then built retaining age, use of metastasis surgery and variables associated with overall survival with a univariate *p*-value <0.1. The management of missing data was performed as described in previous analyses. 

All results are presented as hazard ratios (HR), and all tests were performed with a two-sided alpha risk of 5%. Statistical analyses were performed with R software version 4.0.2. 

## 3. Results

**Figure 1:**
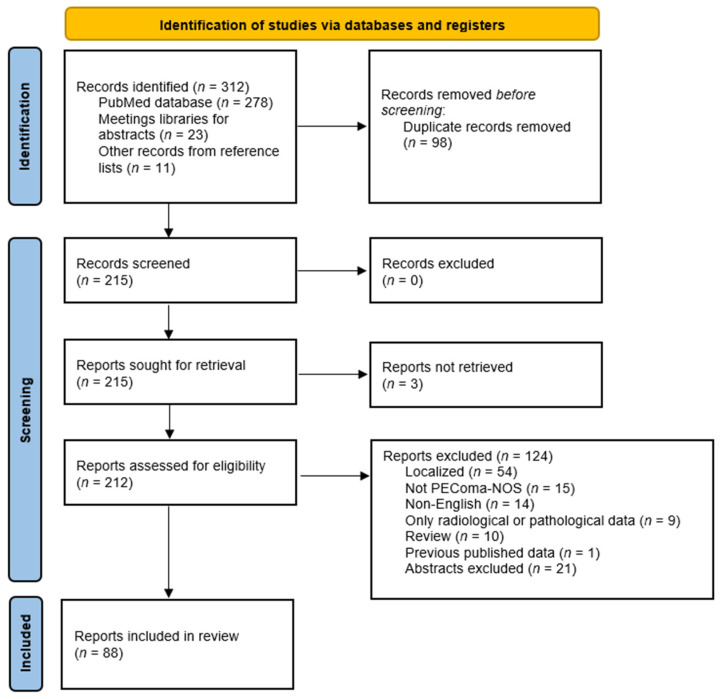
PRISMA flow diagram of the literature search strategy.

Overall, 312 records were identified from the PubMed database, meeting libraries and reference lists search, but only 212 records were eligible (98 duplicate records removed before screening and three not retrieved). Among them, 88 reports fulfilling the inclusion criteria were included in this review (Figure 1). 

**Figure 2:**
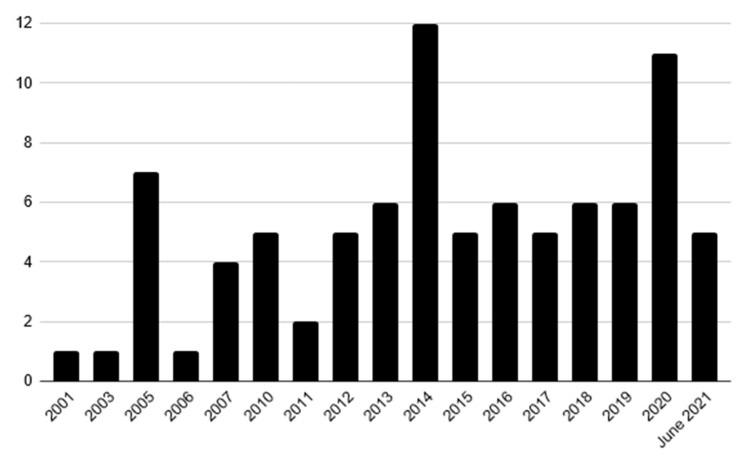
Number of included reports by publication year.

The retained reports were mostly case reports (*n* = 71) [20,21,22,23,24,25,26,27,28,29,30,31,32,33,34,35,36,37,38,39,40,41,42,43,44,45,46,47,48,49,50,51,52,53,54,55,56,57,58,59,60,61,62,63,64,65,66,67,68,69,70,71,72,73,74,75,76,77,78,79,80,81,82,83,84,85,86,87,88,89,90], followed by retrospective case series (*n* = 16) [15,17,91,92,93,94,95,96,97,98,99,100,101,102,103,104] and only one of them was a report of a prospective phase II trial [18]. Details on all the 124 cases reports, case by case are available in Table S1 (Supplementary Materials). Articles were published by 20 different countries but mostly by an American (*n* = 28), followed by a Chinese (*n* = 17) and an Italian (*n* = 10) first author. Less than 30% of articles were published in 2012 or before (*n* = 26), whereas more than 40% were published in the last five years (*n* = 36) (Figure 2). 

### 3.1. Patients and Tumors General Characteristics 

#### 3.1.1. Data Based on Patients’ Cohorts 

Seven cohorts were included in this review, all with different results reported given the variability of collected data between articles [17,18,97,98,101,102,104] (Table 1). The number of patients included in these studies varied between 7 to 50, patients were mostly female with a median age between 47.5 to 67 years old. All but one were observational retrospective studies (mono or multicentric). The only prospective data collected came from the abstract presented at the of 2020 ASCO annual meeting reporting the “AMPECT” phase II trial, which was the first trial to prospectively assess a treatment in advanced malignant PEComa [18].

One study from a sarcoma reference center, collected data on clinical and imaging features of malignant PEComa [98]. Within their cohort of 26 metastatic patients with a median follow-up of 11.5 months, the most common primary tumor location was retroperitoneum (38.9%), followed by female genital tract including the uterus (27.8%) and gastrointestinal tract. Regarding the most common metastatic sites, lungs with 21.6% of all sites ranked first, followed by the liver (17.6%) and the peritoneum (10.8%). Lymph node metastatic sites represented only 9.5% of all sites. TSC features or family history were not found in any of these patients. 

Five studies reported the activity of several systemic treatments but mainly focusing onto mTOR inhibitors. Among the four studies assessing the efficacy of mTOR inhibitors monotherapy, the objective response rate (ORR) was available in three, being around 40%, with no obvious difference across the different available mTOR inhibitors [17,18,97,104]. PFS was only available in two reports and varied from 5.4 to 27.7 months. The updated results of the AMPECT with a longer follow-up, reported that 71% (95% confidence interval (CI): 47.7; 85.1) of the 31 evaluable patients, did not relapse at 6 months of nab-sirolimus treatment. 

Other efficacy data for nab-sirolimus, included a short median time to treatment response of 1.4 months (95%CI: 1.3; 2.8) and a median duration of response not yet reached, as five of the 12 responders were still on treatment after more than two years. One of the most interesting results was the correlation of *TSC1*/*TSC2* mutational status with efficacy. Indeed, among the 25 patients for whom mutational analysis was available, eight out of nine (89%) *TSC2* mutated tumors responded to treatment. Another recent study on a limited number of seven patients, assessed the addition of an anti-estrogen treatment in female patients becoming resistant to mTOR inhibitors [102]. 

After a median follow-up of 13.1 months, ORR was 43%, and the disease control rate was 86% suggesting evidence of a crosstalk between the mTOR pathway and estrogen receptor signaling. Across all studies, the safety profile was as expected from previous studies with dose reduction occurring in 35% of patients and treatment discontinuation in less than 10% of cases. Only one of these retrospective studies reported efficacy data for chemotherapy and VEGF inhibitors [17]. 

Regarding chemotherapy, two protocols were commonly prescribed: anthracycline-based as for other soft tissue sarcomas (STS) and gemcitabine-based chemotherapy. For both regimens, the median PFS was around 3 months with a slightly higher ORR of 20% (95%CI: 4.3; 48.1) for gemcitabine-based chemotherapy compared to the anthracycline-based protocols (ORR: 13% 95%CI: 2.8; 33.6) contrasting with data reported in STS. Efficacy data for VEGF inhibitors (also used for other STS) showed an ORR of 8.3% (95%CI: 0.2; 38.5), lower than reported data for chemotherapy and mTOR inhibitors, whereas the median PFS of 5.4 months was similar to the mTOR results. Among the systemic treatments reported in these studies, one patient was included in a phase I trial and received thalidomide, which did not show any efficacy [97]. 

Interestingly, one of the latest studies investigated, as a main objective, the genomic landscape of malignant PEComa [101]. Tumor samples from 31 different tumors were analyzed with a single integrated DNA and RNA sequencing assay. Not surprisingly, the first genomic alterations over the 100 identified were in *TSC2* (32.3%), *TFE3* (16.1%), *TSC1* (9.6%) and *FLCN* (6.4%). Each tumor had an average of 3.2 genomic alterations, and all *TFE3* alterations were gene fusions. These data confirmed what was previously reported but overall demonstrated the feasibility to run these analyses in routine clinical practice, a conclusion emphasized by the AMPECT trial results.

#### 3.1.2. Clinical Data Based on Individual Case Reports 

Out of the 124 patients’ cases collected (half of them from individual case reports and the other half from case series), 89 (71.8%) were female and 35 (28.2%) were male (Table 2). The median age was 43.5 years old and ranged from 2 to 80. Details on the tumor characteristics are in Table 2.

Most cases had a distant extension, either at diagnosis for 45.2% (*n* = 56) or at relapse for 44.4% (*n* = 55). Among them, 67.4% (*n* = 64) had more than three metastatic lesions, while 32.6% (*n* = 31) were oligometastatic. The first metastatic site was the lungs representing 27.2% (*n* = 41) of all metastatic sites, followed by the lymph nodes (18.5%), liver (17.9%) and peritoneum (13.9%). In addition to the metastatic subpopulation and according to their oncologic history, 8.1% (*n* = 10) of cases included in this review were only treated for a locally advanced tumor at diagnosis and 2.4% (*n* = 3) for a locally advanced relapse.

Follow-up was available for 112 cases and reached the median of 22 months, ranging from one to 240 months. Among the 116 patients for whom a vital status was known at the latest follow-up, 63.8% (*n* = 74) were alive at the latest news whereas 35.3% (*n* = 41) died from the disease. The results presented below were based on this cohort of advanced PEComa patients gathered for the review’s purpose.

### 3.2. Detailed Histopathological Features

The current criteria to assess malignancy were first described by Folpe and Kwiathosky in 2005 [15]. Since then, malignant PEComas have been defined by the presence of at least two of these criteria: tumor size ≥ 5 cm, infiltrative growth pattern, high nuclear grade and cellularity, necrosis, vascular invasion and mitotic rate ≥ 1/50 per HPF. As already mentioned, in 2012 Bleeker et al. suggested a revised set of risk stratification to simplify the use of these criteria by only considering the size and the mitotic activity to stratify the risk of relapse [16].

In this literature review, we focused only on patients with advanced PEComas for whom 93.3% (*n* = 83) were considered malignant according to Folpe’s criteria and 72.2% (*n* = 52) according to Bleeker’s criteria (Table 3). Surprisingly, Bleeker’s risk stratification was more difficult to report and could only be defined for 72 of the 124 PEComas. In our cohort, 91.1% (*n* = 92) of patients had a tumor size of 5 cm or greater and 82.8% (*n* = 53) had a high nuclear grade and/or cellularity. Moreover, 77.0% (*n* = 67) of patients had a PEComa with tumor necrosis, while 64% (*n* = 32), 69.2% (*n* = 45) and 77.1% (*n* = 64) had a tumor with an infiltrative growth pattern, a vascular invasion and a mitotic rate of 1/50 HPF or higher, respectively.

PEComas have immunohistochemically distinctive perivascular epithelioid cells with both melanocytic and smooth muscle expression. Regarding the different IHC performed to assess melanocytic differentiation, HMB45 was more frequently used (*n* = 81) and its expression more often found with 95.1% (*n* = 77) of positive tumors compared to Melan A, with only 72.9% (*n* = 51) of positive tumors. While two smooth muscle differentiation markers were almost used for the same number of tumors (around 65 tumors), smooth muscle actin (SMA) was more expressed with 69.7% (*n* = 46) of positive tumors compared to 55.4% (*n* = 36) of positive tumors for Vimentin/Desmin. 

Regarding TFE3 expression, 16 (59.2%) of the 27 assessed tumors were TFE3 positive. Overexpression of TFE3, member of the microphthalmia transcription factors family (MiTF), mediates the expression of cathepsin-K, which might be another IHC marker helpful to diagnose TFE3-altered PEComas [105,106,107]. In our cohort, Cathepsin K was only assessed on six tumors but all of them were positive (Table 4).

### 3.3. Molecular Features

Among the 124 cases, genomic alterations were assessed in a small fraction of cases. In the great majority of the case reports, there was no information towards molecular status (*n* = 98). *TSC1/2* mutations and *TFE3* translocation were searched in 26 and 14 of cases, respectively (Table 4). In only three cases were both mutations investigated concomitantly. *TSC1/2* mutations with loss of function were detected in 50% (*n* = 13) of all screened cases. Five tumors had a mutation in *TSC1* gene, while eight had one in *TSC2*. A *TFE3* translocation was detected in 64.3% (*n* = 9) of screened cases, but *TFE3* fusion partner was only characterized in one case, diagnosing a PSF-TFE3 translocation. Regarding other genomic alterations, a mutation in *ATRX* gene was reported in one PEComa, whereas no mutation was described in the *FLCN* gene. 

### 3.4. Treatment Strategies

Among the 124 patients included in the case reports cohort, 13 (10.5%) were locally advanced either at diagnosis or relapse, whereas 55 (44.4%) patients had a metastatic relapse, and 56 (45.2%) were already metastatic at the time of diagnosis.

#### 3.4.1. Locally Advanced PEComas

**Figure 3:**
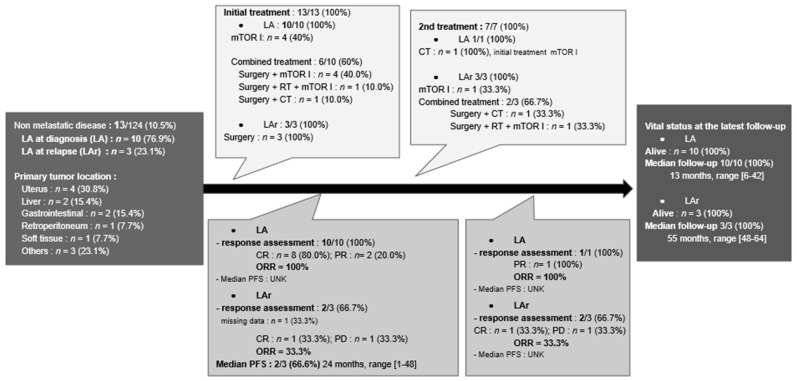
Flowchart of the management for locally advanced PEComas. Abbreviations: CR: complete response as defined by RECIST 1.1 criteria; CT: chemotherapy; DOD: dead of the disease; I: inhibitors; LA: locally advanced at diagnosis, LAr: locally advanced at relapse; ORR: objective response rate; PFS: progression free survival; PR: partial response; RT: radiotherapy; and UNK: unknown.

Among the ten patients who had a locally advanced disease at diagnosis, six of them (60%) were treated by surgery associated with a systemic treatment (Figure 3). mTOR inhibitors were the other initial treatment received by the four remaining patients (40%). With a median follow-up of 13 months (range: 6–42 months), only one patient experienced local progression and received second-line chemotherapy, but all were alive at the latest follow-up. 

After their initial surgery, three patients had a locally advanced relapse in the operating bed with a median delay of 24 months (range: 1–48 months) (Figure 3). Among them, two patients (66.7%) had surgery combined with at least a systemic treatment, and one was treated by mTOR inhibitors alone. All patients were alive at the latest follow-up, with a median follow-up of 55 months (range: 48–64 months). 

#### 3.4.2. Metachronous Metastatic PEComas

**Figure 4:**
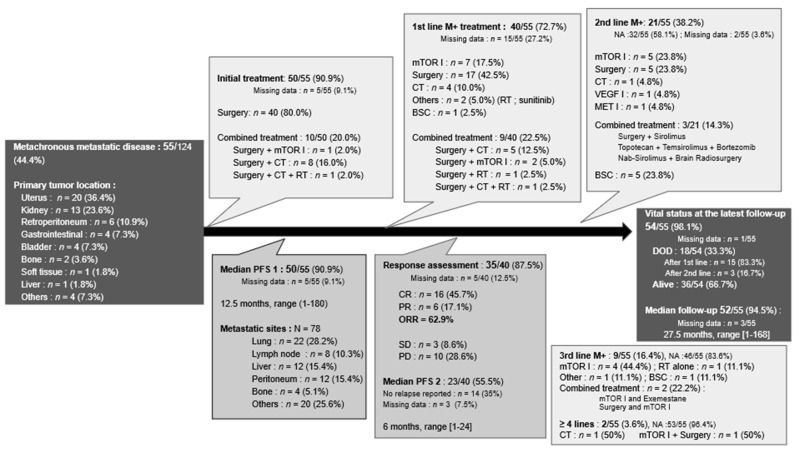
Flowchart of the management for metachronous metastatic PEComas. Abbreviations: BSC: best supportive care; CR: complete response as defined by RECIST 1.1 criteria; CT: chemotherapy; DOD: dead of the disease; I: inhibitors; M+: metastatic; NA: not applicable; ORR: objective response rate; PD: progressive disease; PFS: progression free survival; PR: partial response; RT: radiotherapy; and SD: stable disease.

Among the 55 patients who experienced metastatic relapse, initial treatment for the primary tumor was known in 90.9% (*n* = 50) of cases (Figure 4). The vast majority were treated by surgery alone, while 20% (*n* = 10) underwent surgery associated with another treatment, being mostly chemotherapy. The median PFS1 was 12.5 months (range: 1–180 months). At the time of metastatic relapse, first-line treatments were known for 72.7% (*n* = 40) of patients and were very heterogeneous. 

Surgery was part of the treatment either alone or in combination for 65% (*n* = 26) of patients. mTOR inhibitors were the most used systemic treatment (*n* = 7), followed by chemotherapy (*n* = 4) and VEGF inhibitors (*n* = 1). Metastatic re-progression occurred with a median PFS2 of 6 months (range: 1–24 months). Twenty-one (38.2%) of the 55 patients received second-line treatment, which was mostly systemic treatments (*n* = 9) and, among them, most often mTOR inhibitors alone or in combination. Further lines of treatment were reported for nine patients and are detailed in Figure 4. 

Among all patients with a metachronous metastatic disease, the median follow-up period was 27.5 months (range: 1–168 months). Two thirds (*n* = 36) of patients were alive at the date of the latest news. 

#### 3.4.3. Synchronous Metastatic PEComas

**Figure 5:**
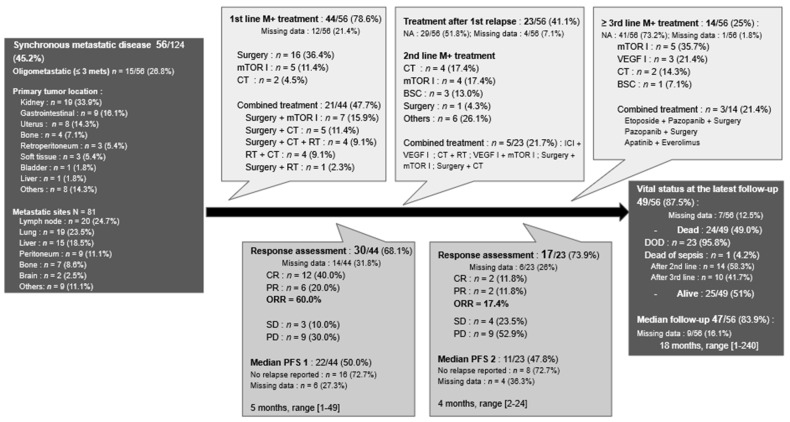
Flowchart of the management of synchronous metastatic PEComas. Abbreviations: BSC: best supportive care; CR: complete response as defined by RECIST 1.1 criteria; CT: chemotherapy; DOD: dead of the disease; I: inhibitors; ICI: immune checkpoint inhibitors; M+: metastatic; mets: metastases; NA: not applicable; ORR: objective response rate; PD: progressive disease; PFS: progression free survival; PR: partial response; RT: radiotherapy; and SD: stable disease.

First-line treatment characteristics were available for 78.6% (*n* = 44) of patients with metastases at diagnosis (Figure 5). Twenty-one patients (47.7%) received a combination of treatments either surgery/radiotherapy with a systemic treatment (*n* = 20) or surgery with radiotherapy (*n* = 1). Surgery was the only treatment in 36.4% (*n* = 16) of cases with different objectives ranging from palliative debulking to curative-intent surgery. Finally, seven patients only received a systemic treatment; either mTOR inhibitors in 11.4% (*n* = 5) or chemotherapy in 4.5% (*n* = 2) of patients. 

The median PFS1 for first-line therapy was 5 months (range: 1–49 months). Data on second-line treatment were reported in 23 (41.1%) of the 56 case reports. Among second-line treatment, five patients (21.7%) had a combination of treatments, four (17.4%) had mTOR inhibitors, and four had chemotherapy, while six (26.1%) had other systemic treatments, mostly VEGF inhibitors. Finally, one patient had palliative surgery, and three only received best supportive care. Based on the available data, the median PFS2 for second-line was 4 months (range: 2–24 months). Data on the third line and beyond were reported in 14 cases (25%) and are detailed in Figure 5. 

Almost half of the patients eventually died, mostly due to their disease (*n* = 23). The median follow-up period for this synchronous metastatic population was 18 months (range: 1–240 months).

### 3.5. Risk Stratification and Outcomes

One of the main objectives of this review was to define risk factors in terms of survival. In the general population of the 124 patients from case reports, the median OS was 60 months (95%CI: 33; NA). This median OS dropped to 28 months for the synchronous metastatic population and increased to 126 months in the metachronous metastatic population. 

#### 3.5.1. In the Whole Population 

In terms of risk factors associated with PFS, age, size ≥ 5 cm, vascular invasion, high nuclear grade, necrosis, infiltrative growth pattern, TFE3 expression, Folpe’s risk category and chemotherapy or mTOR inhibitors in the non-metastatic stage were not significantly associated with an increased risk of relapse in univariate analysis.In contrast two factors were significantly associated with an increased risk of relapse in univariate analysis: mitotic rate ≥ 1/50 HPF (HR: 3.64 95%CI 1.09;12.14, *p* < 0.05) and malignant Bleeker’s grouped-risk category (HR: 3.59 95%CI 1.06;12.13; *p* < 0.05). 

Among variables with a *p*-value < 0.1 included in the multivariate analysis (vascular invasion and necrosis), Bleeker’s grouped-risk category was chosen over the mitotic rate as the composite variable of tumor size and mitotic index. Only one of them was significantly associated with PFS: malignant Bleeker’s grouped-risk category (HR: 8.00 95%CI 1.00; 63.95; *p* = 0.0498). Due to a lack of events, surgery and radiotherapy performed in non-metastatic stages could not be tested. 

In terms of risk factors associated with OS based on univariate analysis, the presence of metastasis at diagnosis was significantly associated with an increased risk of death (HR: 2.19; 95%CI 1.17;4.09; *p* = 0.014), as was malignant Bleeker’s grouped-risk category (HR: 4.82; 95%CI 1.13;20.58; *p* = 0.034) (Table 5). On the contrary, age, vascular invasion, high nuclear grade, mitotic rate ≥ 1/50 HPF, necrosis, infiltrative growth pattern, TFE3 expression and type of treatment at diagnosis (surgery alone vs. surgery + adjuvant therapy) were not significantly associated with OS, even if there was a trend towards a poorer prognosis for histological factors. Due to a lack of events, primary tumor site, size ≥ 5 cm, Folpe’s and Bleeker’s risk categories and type of systemic treatment at diagnosis (none vs. chemotherapy vs. mTOR inhibitors) could not be tested. 

**Figure 6:**
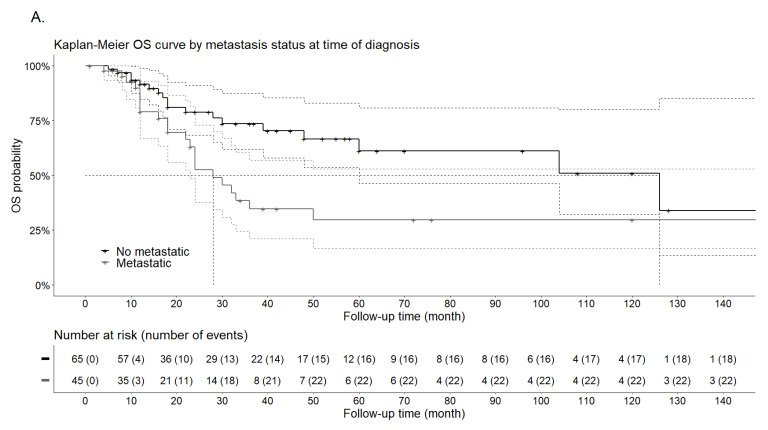

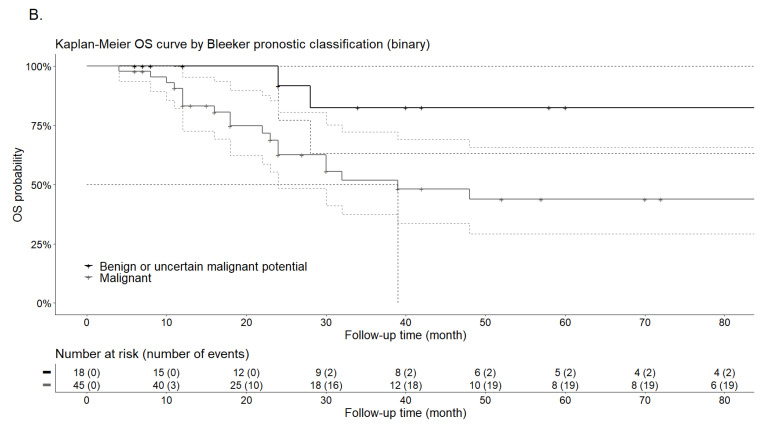
Kaplan–Meier OS curves by metastatic status at diagnosis (**A**) and by grouped -Bleeker classification (**B**).

In multivariate analysis, the presence of metastasis at diagnosis (HR: 2.59, 95%CI 1.06; 6.33, *p* < 0.036) and the malignant Bleeker’s grouped-risk category (HR: 4.66; 95%CI 1.07; 20.19; *p* = 0.039) were also significantly associated with an increased risk of death (Figure 6).

#### 3.5.2. Focus on the Metastatic Population 

In this subgroup of metastatic patients, none of the tested variables in univariate analysis was significantly associated with the OS (Table 6). As planned, factors with a *p*-value lower than 0.1 were included in the multivariate analysis: presence of metastasis at diagnosis, presence of more than three metastasis, presence of lymph node metastasis and liver metastasis. Among them, the presence of lymph node metastasis was associated with an increased risk of death (HR: 3.11; 95%CI 1.13; 8.60, *p* < 0.05). This risk factor has to be considered carefully because of the non-significant univariate analysis results. 

Regarding the type of first metastatic treatment received, none of them increased or decreased significantly in OS in the univariate analysis. In order to define a best treatment sequence, a direct comparison of chemotherapy with mTOR inhibitors adjusted on the use or not of surgery was also performed. Patients included in this analysis (*n* = 42) were those who received systemic treatment as first-line treatment of their metastatic disease. The median OS was 33 months (range: 18 months–NA) for chemotherapy and 23 months (range: 18 months–NA) for mTOR inhibitors (HR: 1.51; 95%CI 0.66; 3.43, *p* = 0.33). No conclusion could be drawn from this analysis due to a lack of power.

## 4. Discussion

A number of articles have been published on PEComas in the last ten years showing an increasing interest for a better understanding of this very heterogeneous and still poorly understood family of tumors. There has been a recent effort to publish multicenter retrospective case series instead of isolated case reports, in order to homogenize data collection and to run more reliable statistical analyses. 

Updated results from the first clinical trial ever on PEComa were recently presented and brought new insights on PEComas biology and treatments. However, even though more data have been published since 2012 and the latest review on NOS-PEComas, it is striking that there is still a need for reporting valuable data, such as genomic alterations. Few molecular alterations can be found in NOS-PEComas, supporting a global effort to retrieve and report these molecular characteristics in addition to clinical outcomes, as they may be hypothesis-generating data.

In this review, clinical features were very similar to previously reported data, with a strong prevalence of female patients and a quite low median age of 43.5 years [15,16,28]. Even though the most frequent anatomic tumor locations were kidneys, the uterus and the gastrointestinal tract, the reported diversity of primary tumor locations corroborate the fact that these tumors can arise from any organs. This also reflects that less PEComas are misdiagnosed thanks to the pathology community and their network of expert pathologists on soft tissue tumors reviewing most cases [108,109]. This expert pathologist’s review is especially necessary in case of a late relapse, as some PEComas were initially considered as other entities.

Risk categories, even if not explicitly reported by authors, were easier to determine posteriori using the Folpe’s method than the Bleeker’s criteria. Indeed, missing data for tumor size and/or mitotic rate made Bleeker’s classification impossible. However, being able to retrieve only two among the six high risk features according to Folpe’s classification was enough to classify the tumor as a malignant PEComa, even though other features may be missing. 

Interestingly, Bleeker et al. originally proposed a revised set of risk stratification, to improve the number of reported risk categories in future published data since they had to deal with a great deal of missing data in particular with Folpe’s criteria. This review showed no improvement in the exhaustivity of reported data, although these prognostic criteria have now been assessed on different cohorts and should be adopted by the community. 

In terms of pathology and IHC markers, the review showed that the two melanocytic and myofibroblastic components were always assessed. At the light of the reported data, HMB45, Melan A and smooth muscle actin (SMA) appeared to be the most consensual markers. More recently, Cathepsin K was reported as a potential new useful IHC marker since it is expressed in most visceral PEComas [106]. Cathepsin K is a protease involved in the osteoclast function and that is regulated by proteins from the MiTF family, which includes TFE3 [105]. 

This marker could be as specific as HMB45 and interesting to better discriminate PEComas from other differential diagnoses (melanomas, STS etc.). Despite its potential, Cathepsin K was only reported in six cases among the 128 analyzed. TFE3 expression assessed by IHC was slightly more frequently reported, but not enough to speculate on its correlation with other features or survival. As previously reported, TFE3 immunoreactivity has to be cautiously interpreted since a high sensitivity of the assay can result in enhanced TFE3 detection because of its ubiquitous low expression. Hence, IHC should be performed to identify positive PEComas before looking for a *TFE3* gene rearrangement, since some of these PEComas will have a *TFE3* gene fusion [93]. 

As already mentioned, genomic alterations were reported in a very limited number of case reports. The published most valuable dataset on molecular status owing to new techniques came from a case series by Akumulla et al., but the data were not correlated to clinical behavior or response treatment [101]. Even though access to molecular biology has increased since previous reviews, very few cases reported the search for a *TFE3* translocation, a *TSC1* or *TSC2* loss of function mutations or even no *FCLN* mutation, considered to be the more recent genomic alterations described in PEComas. 

The only information reported by many case reports was the notification of a “no TSC profile” based only on clinical features and family history. Yet, genomic *TSC2* and *TSC1* alterations are frequent and associated with PEComa pathogenesis [110], inducing proliferation as they activate mTOR pathway [111]. Moreover, the most recent data showed that PEComa patients harboring *TSC2* mutations and treated with mTOR inhibitors have a better ORR and PFS as compared to those with *TSC1* mutations. Translational studies are warranted in the light of these findings as well as for tumors progressing after mTOR inhibitors, where resistance mutations may occur. 

The second important pathway in PEComa pathogenesis involves *TFE3* translocations [93]. In the literature, different partners of *TFE3* have been described and keep on being described. The presence of the *TFE3* fusion protein probably substitutes MiTF in these PEComas, explaining the absence of MiTF expression and the lower expression of Melan A. *TFE3* rearrangements and *TSC1/2* alterations were, for a long time, considered to be mutually exclusive [10], but recently a case of TCS1-mutated PEComa displaying a TFE3-altered phenotype was reported, challenging this conclusion [112]. Hence, developing molecular biology analysis in PEComas is mandatory both to decipher the tumor pathogenesis and to select for the most relevant therapy. 

Based on the knowledge of mTOR pathway activation in other primary cancers, the addition of hormonal therapy was assessed in a small case series of progressive PEComas after mTOR inhibitors and showed an interesting efficacy signal. Recently, a subdivision into two molecular subgroups of PEComas was proposed: type 1, responding to mTOR inhibitors and type 2 responding to c-MET inhibitors [113]. c-MET inhibitors could be more efficient in *TFE3*-altered PEComas, since *TFE3* fusions activate MET signaling by transcriptional up-regulation [114]. In this review of the literature, due to missing data, it was impossible to conclude on the optimal treatment sequence between chemotherapy and mTOR inhibitors.

Among systemic therapies, mTOR inhibitors are by far the most frequently used across the different lines either as a curative intent treatment combined with a radical treatment (surgery or radiotherapy) or in the palliative setting. However, as of today, there is no clear recommendation, and the question remains to be answered of whether it can or cannot be considered as the gold standard for all patients or only for a subgroup of patients, such as *TSC2*-mutated patients. 

In an attempt to predict the response to mTOR inhibitors, an interesting study showed that the level of phosphorylated S6 ribosomal protein expression, reflecting the mTOR pathway activation, was predictive of early tumor response to the drug [115]. However, there is no study assessing the response to mTOR inhibitors for PEComas with and without *TFE3* translocation. Although mTOR inhibitors, as a family, appear to have similar efficacy and toxicity spectra, the only prospective data investigated nab-sirolimus, which could privilege its prescription over the others. However, there are issues regarding nab-sirolimus approval and reimbursement, and the drug requires closer monitoring as compared to other mTOR inhibitors. 

In addition, very little data has been published on treatment strategies, in particular on the preferred treatment sequences or the benefits of surgery in oligometastatic patients. However, several case reports argue on the benefit of a neoadjuvant treatment in locally advanced PEComa to be able to perform a conservative surgery, such as a fertility sparing surgery. 

The median OS observed in the whole population was 60 months, an unexpectedly long survival, which may be explained by (i) the inclusion of 10.5% non-metastatic patients and (ii) the fact that 44.4% patients were not metastatic at diagnosis. Moreover, the estimate of this median survival is rather imprecise, due to a large number of patients censored early in the study and the wide range of the CI95% [33-not reached]. Despite the efforts to present a reliable estimation of the risk, the small number of events in several tested variables certainly affected this objective. 

Interestingly, even with the limited available data, a malignant tumor as defined by Bleeker’s risk category was confirmed as a poor prognostic factor in this review, provided that the two other risk categories were grouped together. In addition to this factor and as expected, metastatic disease at diagnosis was the main prognostic factor associated with a shorter survival. Once again, these results have to be interpreted with caution, since there might be a potential attrition bias in both survival analyses of the whole population. 

Indeed, patients excluded from the analyses due to missing data more frequently had tumors with vascular invasion and necrosis. The multiple imputation technique was considered to reduce this limit but was judged unreliable with regard to the large proportions of missing data on certain variables. When we assessed survival in the metastatic subpopulation, lymph nodes metastases appeared to confer a worse prognosis to patients, probably reflecting the aggressiveness of tumors, which, as other soft tissue tumors, usually do not spread through the lymph vessels. 

In addition to the study limitations that have been mentioned above in the text, retrospective reviews with case reports may erroneously favor case reports with infrequent outcomes and insufficient follow-up, thus, not reflecting the usual natural history of the disease. Finally, by restricting the search to (“metastatic” OR “metastasis” OR locally advanced”), we might have missed some case reports on locally advanced PEComa-NOS that could have been described only as a localized disease. Even with these queries, we still had to exclude 54 articles, because they concerned localized PEComa without any systemic treatment during their therapeutic management. Thus, data on locally advanced PEComas should be interpreted with caution also due to their low representation in this review.

## 5. Conclusions

This systematic review of the literature provides an overview of the natural history and the therapeutic management of advanced PEComas. Unfortunately, due to a significant lack of data, significant conclusions cannot be drawn on risk stratification even on well-known high-risk pathologic factors. We propose that working on appropriate, convenient and reliable risk stratification with pathology experts is warranted to homogenize and facilitate both diagnosis and risk-classification of these rare tumors. Similarly, minimum and systematic standards for molecular biology assessments should also be implemented to drive the use of targeted therapies. These collaborative efforts are highly anticipated to run international prospective trials in rare tumors, such as PEComas. 

## Figures and Tables

**Table 1:** Clinical and outcome data from patient cohorts.

References	Metastatic Patients, *n* (%)	Median Age, Years [Range]	Female Patients, %	Main Primary Tumor Location, %		PEComa-NOS Subtype, %	Main Metastatic Sites, %			Median Follow-Up, Mos [Range]	ORR, % [95%CI]					Median PFS, Mos [95%CI] or (IQR)				
				Kidney	Uterus		Lung	Lymph Node	Liver		Antra-Based CT	Gem-Based CT	mTOR inh	VEGF inh	Others	Antra-Based CT	Gem-Based CT	mTOR inh	VEGF inh	Others
Benson et al., 2014 [97]	10 (100)	47.5 [26–63]	80.0	30.0	20.0	100	-	0	-	22.8 [–]	-	NA	-	NA	0 °	-	NA	-	NA	0 °
Tirumani et al., 2014 [98]	26 (72.2)	53.1 [35–77]	72.0	22.2	22.2	100	21.6	9.5	17.6	11.5 [0–75]	NA	NA	NA	NA	NA	NA	NA	NA	NA	NA
Sanfilippo et al., 2019 [17]	50 (94.3)	54 [26–76]	69.8	11.3	20.8	79.2	9.4	22.6	17.0	30.1 [9.1–58.9]	13 [2.8–33.6]	20 [4.3–48.1]	41 [25.6–57.9]	8.3 [0.2–38.5]	NA	3.4 (2.3–4.9)	9 (4.4–39.4)	5.4 (2.5–9.6)	5.4 (2.5–9.6)	NA
Wagner et al., 2020 [18]	31 (91.2)	-	-	-	-	100	-	-	-	14.5 [–]	NA	NA	39 [21.8–57.8]	NA	NA	NA	NA	-	NA	NA
Akumalla et al., 2020 [101]	31 (100)	50 [8–76]	48.0	3.2	3.2	100	-	-	-	NA	NA	NA	NA	NA	NA	NA	NA	NA	NA	NA
Sanfilippo et al., 2020 [102]	*	67 [47–76]	100	0	72	100	57.0	-	29.0	13.1 [–]	NA	NA	NA	NA	43 [16.0–75.0] #	NA	NA	NA	NA	9.8 [3.7–NR] #
Lin et al., 2021 [104]	13 (76.5)	-	-	-	-	100	-	-	-	-	NA	NA	45.5 [–]	NA	NA	NA	NA	27.7 [4.7–50.5]	NA	NA

* *N* = 7 locally advanced and metastatic patients without any more details. Abbreviations: Antra: antracycline; CI: confidence interval; CT: chemotherapy; Gem: gemcitabine; IQR: interquartile range; mos: months; NA: not applicable; NR: not reached; ORR: objective response rate; PEComa-NOS: perivascular epithelioid cell neoplasm-not other specified; PFS: progression free survival; yrs: years; –: missing data; °: thalidomide; and #: association mTOR inhibitor and anti-estrogen.

**Table 2:** Patients and tumor characteristics from individual case reports (*N* = 124).

Population Characteristics	*n*	%
Median age years (mean, range) *	43.5 (43, 2–80)	
<30 years	29	23.4
30 < 45 years	35	28.2
45 < 60 years	35	28.2
≥60 years	23	18.5
Sex		
Female	89	71.8
Male	35	28.2
Primary tumor location		
Kidney	32	25.8
Uterus	32	25.8
Gastrointestinal	15	12.1
Retroperitoneum	10	8.1
Bones	6	4.8
Bladder	5	4.0
Soft tissue	5	4.0
Liver	4	3.2
Others	15	12.1
Tumor size ^§^		
Median cm (mean, range)	10 (11.8, 2.4–37)	
<5 cm	9	8.9
≥5 cm	92	91.1
Histotypes		
PEComa-NOS	101	81.4
Epithelioid AML	23	18.5
Worse disease extension		
Locally advanced	10	8.1
Locally advanced relapse	3	2.4
Synchronous metastases	56	45.2
Metachronous metastases	55	44.4
Number of metastatic lesions ^$^		
≤3	31	32.6
>3	64	67.4
Metastatic sites		
Lung	41	27.2
Lymph node	28	18.5
Liver	27	17.9
Peritoneum	21	13.9
Bone	11	7.3
Others	23	15.2
Median follow up, months (mean, range) ^‡^	22 (32.1, 1–240)	
Vital status at the latest follow-up ^#^		
Alive	74	63.8
Dead of the disease	41	35.3
Dead from another cause	1	0.8

* *n* = 122 (2 missing data), ^§^ *n* = 94 (30 missing data) for median data and *n* = 101 (23 missing data) for size categories, ^$^ *n*= 95 patients (111 patients in the metastatic sub-population but 16 missing data), ^‡^ *n* = 112 (12 missing data), and ^#^ *n* = 116 (8 missing data). Abbreviations: AML; angiomyolipoma; and PEComa-NOS: perivascular epithelioid cell neoplasm-not other specified.

**Table 3:** Details on high-risk features described as Folpe’s criteria and Folpe and Bleeker’s risk classification from individual case reports (*N* = 124).

High Risk Histologic Factors	*n*	%
Size ≥ 5 cm *		
Present	92	91.1
Absent	9	8.9
Infiltrative growth pattern ^&^		
Present	32	64
Absent	18	36
High nuclear grade ^µ^		
Present	53	82.8
Absent	11	17.2
Mitotic rate ≥ 1/50HPF ^p^		
Present	64	77.1
Absent	19	22.9
Necrosis °		
Present	67	77.0
Absent	20	23.0
Vascular invasion ^£^		
Present	45	69.2
Absent	20	30.8
Folpe’s risk category ^$^		
Benign	2	2.2
Uncertain malignant potential	4	4.5
Malignant	83	93.3
Bleeker’s risk category ^#^		
Benign	2	2.8
Uncertain malignant potential	18	25.0
Malignant	52	72.2

* *n* = 101 (23 missing data), ^&^ *n* = 50 (74 missing data), ^µ^ *n* = 64 (60 missing data), ^p^ *n* = 83 (41 missing data), ° *n* = 87 (37 missing data), ^£^ *n* = 65 (59 missing data), ^$^ *n*= 89 patients (35 missing data), and ^#^ *n* = 72 (52 missing data).

**Table 4:** Immunohistochemistry profile and major genomic alterations from individual case reports (*N* = 124).

IHC and Molecular Markers	*n*	%
IHC		
HMB45 ^&^		
Present	77	95.1
Absent	4	4.9
Melan A ^µ^		
Present	51	72.9
Absent	19	27.1
Vimentin/desmin ^p^		
Present	36	55.4
Absent	29	44.6
SMA °		
Present	46	69.7
Absent	20	30.3
TFE3 ^£^		
Present	16	59.2
Absent	11	40.8
Cathepsin K ^$^		
Present	6	100
Absent	0	0
Genomic alteration		
*TSC ^#^*		
*TSC1*	5	19.2
*TSC2*	8	30.8
Absent	13	50.0
*TFE3* translocation “		
Present	9	64.3
Absent	5	35.7
Others	1 (ATRX)	100

* ATRX mutation. ^&^ *n* = 81 (43 missing data), ^µ^ *n* = 70 (54 missing data), ^p^ *n* = 65 (59 missing data), ° *n* = 66 (58 missing data), ^£^ *n* = 27 (97 missing data), ^$^ *n* = 6 patients (118 missing data), ^#^ *n* = 26 (98 missing data), and “ *n* = 14 (110 missing data). Abbreviations: IHC: immunohistochemistry; SMA: Smooth Muscle Actin; and *TSC1/2*: Tuberous Sclerosis Complex 1/2.

**Table 5:** Univariate and multivariate analyses of clinical and histologic risk factors related to OS in the whole cohort population (*N* = 124). Multivariate analysis on 63 patients.

Variable	Mortality Rate *n*/*N* (%)	Median OS (Months) [95%CI]	Univariate Analysis HR [95%CI]	*p*-Value	Multivariate Analysis HR [95% CI]	*p*-Value
	Metastasis at diagnosis					
Absent	18/65 (35)	126 [60; NR]	1	-	1	-
Present	22/45 (49)	28 [23; NR]	2.19 [1.17;4.09]	0.014	2.59 [1.06; 6.33]	0.036
Bleeker’s grouped- risk category						
Benign or uncertain malignant potential	2/18 (11)		1	-	1	-
Malignant	21/45 (47)	39 [24; NR]	4.82 [1.13; 20.58]	0.034	4.66 [1.07; 20.19]	0.039
Tumor ≥ 5 cm						
Absent	0/7 (0)		1	-	-	-
Present	30/82 (37)	104 [32; NR]	NA	-	Not used	-
Mitotic rate ≥ 1/50 HPF						
Absent	4/16 (25)		1	-	-	-
Present	23/57 (40)	32 [24; NR]	2.47 [0.85; 7.19]	0.097	Not used	-
Vascular invasion						
Absent	6/19 (32)		1	-	-	-
Present	15/39 (38)	60 [30; NR]	1.44 [0.56; 3.73]	0.450	Not used	-
High nuclear grade						
Absent	1/9 (11)		1	-	-	-
Present	17/49 (35)	104 [32; NR]	3.99 [0.52; 30.50]	0.183	Not used	-
Necrosis						
Absent	3/19 (15)		1	-	-	-
Present	25/57 (44)	48 [24; NR]	2.65 [0.80; 8.78]	0.112	Not used	-
Infiltrative growth pattern						
Absent	5/16 (31)		1	-	-	-
Present	11/26 (42)	24 [18; NR]	2.02 [0.69; 5.87]	0.199	Not used	-

Abbreviations: CI: confidence interval; HR: Hazard ratio; M+: metastatic; NR: Not reached; NA: Not applicable; and OS: overall survival.

**Table 6:** Univariate and multivariate analyses of metastases features and type of first line treatment in the metastatic population related to OS (*N* = 111). Multivariate analyses on 65 patients.

Variable	Mortality Rate *n*/*N* (%)	Median OS (Months) [95%CI]	Univariate Analysis HR [95%CI]	*p*-Value	Multivariate Analysis HR [95% CI]	*p*-Value
Metastasis at diagnosis						
Absent	18/52 (34.6)	104 [48; NR]	1	-	1	-
Present	22/45 (48.9)	28 [23; NR]	1.84 [0.98; 3.44]	0.056	1.49 [0.59; 3.77]	0.397
Lung metastasis						
Absent	20/46 (43.5)	30 [24; NR]	1	-	-	-
Present	16/43 (37.2)	126 [39; NR]	0.77 [0.40; 1.50]	0.442	Not used	-
Liver metastasis						
Absent	22/65 (33.8)	104 [36; NR]	1	-	1	-
Present	13/23 (56.5)	24 [18; NR]	1.86 [0.93; 3.71]	0.079	1.85 [0.63; 5.45]	0.265
Lymph node metastasis						
Absent	21/63 (33.3)	60 [39; NR]	1	-	1	-
Present	14/24 (58.3)	24 [18; NR]	1.94 [0.98; 3.83]	0.057	3.11 [1.13; 8.60]	0.028
Number of metastases						
0 to 3	5/26 (19.2)	NA [48; NR]	1	-	1	-
> 3	25/55 (45.5)	32 [23; NR]	2.44 [0.93; 6.40]	0.069	2.09 [0.70; 6.29]	0.189
First line M+ treatment = Surgery						
No	10/20 (50.0)	36 [12; NR]	1	-	-	-
Yes	16/52 (30.8)	126 [30; NR]	0.52 [0.24; 1.16]	0.110	Not used	-
First line M+ treatment = Chemotherapy						
No	15/49 (30.6)	126 [36; NR]	1	-	-	-
Yes	11/23 (47.8)	33 [18; NR]	1.84 [0.83; 4.05]	0.132	Not used	-
First line M+ treatment = mTOR inhibitors						
No	14/53 (26.4)	126 [126; NR]	1	-	-	-
Yes	12/19 (63.2)	23 [18; NR]	3.10 [1.42; 6.78]	0.458	Not used	-

Abbreviations: CI: confidence interval; HR: Hazard ratio; M+: metastatic; NR: Not reached; NA: Not applicable; and OS: overall survival.

## Supplementary Materials

The following are available online at https://www.mdpi.com/article/10.3390/cancers13205227/s1, Table S1: List of the 124 reported cases of advanced PEComa-NOS and epithelioid angiomyolipomas.

## Abbreviations

AML	angiomyolipoma
ASCO	American society of clinical oncology
CCMT	clear cell myomelanocytic tumor of the falciform ligament/ligamentum teres
CCST	clear cell sugar tumor of the lung
CI	confidence interval
CTOS	connective tissue oncology society
ESMO	European society of medical oncology
HR	hazard ratio
IHC	immunohistochemistry
LAM	lymphangioleiomyomatosis
MiTF	microphthalmia transcription factors family
NOS	not-otherwise specified
PEComa	perivascular epithelioid cell neoplasm
PFS	progression free survival
ORR	objective response rate
OS	overall survival
STS	soft tissue sarcomas
TSC	tuberous sclerosis complex
WHO	World Health Organization
