# Supplementary material for: Natural History and Treatment Strategies of Advanced PEComas: A Systematic Review
Source: Cancers (Basel). 2021 Oct 18;13(20):5227. doi: 10.3390/cancers13205227 (PMC8533842; doi:10.3390/cancers13205227)
Supplement: Supplementary file 1 [file cancers-13-05227-s001.zip › cancers-1395987-supplementary.pdf]

cancers

MDPI

Natural History and Treatment Strategies of Advanced PEComas: A Systematic Review

Agathe Bourgmayer, Simon Nannini, Paul Bonjean, Jean-Emmanuel Kurtz, Gabriel G. Malouf and Justine Gantzer.

Table S1. List of the 124 reported cases of advanced PEComa-NOS and epithelioid angiomyolipomas.

References	Publication Years	Age	Sex	Tumor size (cm)	Primary tumor location	Risk category (Folpe criteria)	Metastatic status	Treatment of local disease	Time before first mets (mos)	Metastatic sites	1st line metastatic treatment	Time before progression (mos)	2nd line metastatic treatment	Last known status	Follow-up (mos)
Bonetti *et al.*[91]	2001	28	F	9.0	GI	-	Synch M+	NA	NA	LN	Debulking surgery LN+	28	BSC	DOD	28
Bonetti *et al.*[91]	2001	19	F	5.5	Kidney	-	Synch M+	NA	NA	LN	Debulking surgery + CT (AI) + RT	10	BSC	DOD	18
Bonetti *et al.*[91]	2001	41	F	6.0	Kidney	-	Synch M+	NA	NA	Ovary	Surgery	CR	NA	Alive	6
Dimmler *et al.*[20]	2003	68	F	4.0	Uterus	Malignant	Meta M+	Surgery	84	Lung	Wedge of 9 pulmonary mets	CR	NA	Alive	96
Folpe *et al.*[15]	2005	40	M	22.0	GI	Malignant	Meta M+	Surgery	24	Peritoneum; LN	-	-	-	Alive	24
Folpe *et al.*[15]	2005	46	F	12.0	GI	Malignant	Meta M+	Surgery + CT	22	Peritoneum; LN	-	-	-	Alive	27
Folpe *et al.*[15]	2005	59	F	14.5	Uterus	Malignant	Meta M+	Surgery + CT	30	Lung; Liver	-	-	-	Alive	30
Folpe *et al.*[15]	2005	56	F	9.0	Uterus	Malignant	Meta M+	Surgery, RT + CT	11	Lung; Bones	-	-	-	Alive	11
Folpe *et al.*[15]	2005	36	F	-	Uterus	Malignant	Meta M+	Surgery + CT	12	Lung	-	24	-	DOD	39
Jeon *et al.*[21]	2005	9	F	6.5	Uterus	Malignant	Synch M+	NA	NA	LN	IVA 2 cy + Surgery + 6	CR	NA	Alive	34

											cy IVA + RT				
Fukunaga *et al.*[22]	2005	40	F	30.0	Uterus	Malignant	Synch M+	NA	NA	Peritoneum	Surgery + CT + RT	16	-	DOD	16
Evert *et al.*[23]	2005	56	F	8.0	GI	Malignant	Synch M+	NA	NA	Lung	Surgery of the primary location	-	-	Alive	-
Yu *et al.*[24]	2005	12	F	-	Kidney	Malignant	Synch M+	NA	NA	Lung; LN	CT	-	-	DOD	9
Chen *et al.*[25]	2005	16	F	27.0	GI	Malignant	Synch M+	NA	NA	LN	Debulking surgery + CT	2	-	Alive	4
Rigby *et al.*[26]	2005	11	F	10.5	Kidney	Malignant	Synch M+	NA	NA	LN; Peritoneum	Deticene, Carmustine, Vincristine	1	Imatinib	DOD	-
Parfitt *et al.*[27]	2006	53	F	4.8	Adrenal Gland	-	Synch M+	NA	NA	Lung	-	-	-	-	-
Armah *et al.*[28]	2007	59	F	6.0	Uterus	Malignant	Meta M+	Surgery	84	Lung; Kidney	Surgery of all localisations	CR	NA	Alive	15
Park *et al.*[92]	2007	69	M	13.0	Kidney	Malignant	Meta M+	Surgery	8	Liver; Peritoneum	-	-	-	Alive	10
Park *et al.*[92]	2007	46	F	17.0	Kidney	-	Meta M+	Surgery	12	LN; Liver; Peritoneum	-	-	-	Alive	16
Osei *et al.*[29]	2007	49	F	5.3	Soft Tissue	Malignant	Meta M+	RT + CT (AI) + Surgery	13	Lung	Surgery of lung mets	CR	-	Alive	24
El Jack *et al.*[30]	2007	38	M	13.0	Kidney	Benign	Meta M+	Surgery	10	Liver	Surgery	6	-	Alive	16
Wagner *et al.*[31]	2010	65	M	20.0	Retroperitoneum	Malignant	Meta M+	Surgery (tumoral effraction)	24	Retroperitoneum	Surgery	3	MET I > Sirolimus	Alive	52
Wagner *et al.*[31]	2010	70	M	9.0	Kidney	Malignant	LA relapse	Surgery	60	Local relapse	Surgery	12	Sunitinib > Sirolimus	Alive	-

Wagner *et al.*[31]	2010	61	F	9.0	Uterus	Malignant	Synch M+	NA	NA	Lung	Sirolimus	3	Sirolimus > Sorafenib	DOD	8
Italiano *et al.*[32]	2010	55	F	-	Uterus	-	Meta M+	Surgery	180	Lung; Heart	Surgery	9	CT (AI) + mTOR I	Alive	-
Italiano *et al.*[32]	2010	69	F	-	Uterus	-	Meta M+	Surgery	-	Lung	Temsirolimus + Surgery	-	-	Alive	9
Yamashita *et al.*[94]	2010	35	M	-	Bones	Malignant	Synch M+	NA	NA	Bones	RT-CT	9	BSC	Alive	12
Yamashita *et al.*[94]	2010	42	F	-	Uterus	Malignant	Meta M+	Surgery	12	Bones	Surgery of Bone mets + CT	10	BSC	DOD	22
Subbiah *et al.*[33]	2010	58	F	17.0	Retroperitoneum	-	Meta M+	Surgery + CT (Anthra)	24	Liver	Hepatectomy + RT + CT (Anthra)	24	Topotecan + Temsirolimus + Bortezomib	Alive	52
Argani *et al.*[93]	2010	40	F	-	Uterus	-	Meta M+	NA	NA	Lung	-	-	-	-	-
Ross *et al.*[34]	2011	46	F	-	Large right broad ligament	Malignant	Meta M+	Surgery	2	Pelvis	Palliative RT	10	Sirolimus	Alive	36
Nese *et al.*[95]	2011	24	F	-	Kidney	Malignant	Synch M+	NA	NA	Liver; Pelvis	-	-	-	DOD	12
Nese *et al.*[95]	2011	29	M	-	Kidney	Malignant	Meta M+	-	18	Lung; Liver	-	-	-	DOD	18
Nese *et al.*[95]	2011	14	M	11.0	Kidney	Malignant	Synch M+	NA	NA	LN	-	-	-	Alive	240
Nese *et al.*[95]	2011	59	F	34.0	Kidney	Malignant	Synch M+	NA	NA	Colon	-	-	-	-	-
Nese *et al.*[95]	2011	25	F	8.0	Kidney	Malignant	Synch M+	NA	NA	Lung; LN; Liver; Peritoneum	-	-	-	DOD	12
Nese *et al.*[95]	2011	36	M	28.0	Kidney	Malignant	Synch M+	NA	NA	Lung; Liver; Peritoneum	-	-	-	DOD	-
Nese *et al.*[95]	2011	67	M	15.0	Kidney	Malignant	Synch M+	NA	NA	LN	-	-	-	-	-

Nese *et al.[95]*	2011	69	M	13.0	Kidney	Malignant	Meta M+	-	8	LN; Liver	-	-	-	DOD	28
Nese *et al.[95]*	2011	46	F	17.0	Kidney	Malignant	Meta M+	-	12	Liver; Peritoneum	-	-	-	Alive	16
Nese *et al.[95]*	2011	36	M	29.0	Kidney	Malignant	Synch M+	NA	NA	Lung; Liver	-	-	-	DOD	4
Nese *et al.[95]*	2011	58	M	37.0	Kidney	Malignant	Synch M+	NA	NA	LN; Liver	-	-	-	DOD	24
Nese *et al.[95]*	2011	27	M	11.0	Kidney	Malignant	Synch M+	NA	NA	LN	-	-	-	DOD	24
Nese *et al.[95]*	2011	29	M	27.0	Kidney	Malignant	Synch M+	NA	NA	Liver	-	-	-	DOD	11
Nese *et al.[95]*	2011	55	F	12.8	Kidney	Malignant	Synch M+	NA	NA	LN	-	-	-	DOD	12
Nese *et al.[95]*	2011	57	M	4.5	Kidney	Benign	Meta M+	-	58	LN	-	-	-	Alive	58
Gennatas *et al.[35]*	2012	63	F	-	Retroperitoneum	-	Meta M+	Surgery	12	Local relapse; Retroperitoneum	Surgery	4	Imatinib > Everolimus	Alive	37
Kazzaz *et al.[36]*	2012	26	M	-	Bones	Uncertain malignant potential	Synch M+	NA	NA	Lung; Bones	Palliative surgery	5	-	-	-
Desy *et al.[37]*	2012	29	M	5.0	Bones	Malignant	Meta M+	Surgery	2	Lung	Temsirolimus	8	BSC	DOD	10
Niu *et al.[38]*	2012	33	F	-	Cardiac	Malignant	Meta M+	Surgery	8	Local relapse	Surgery + RT	24	mTOR I	Alive	33
Alaggio *et al.[39]*	2012	2	F	12.0	Right umbilical ligament	Malignant	Synch M+	NA	NA	Peritoneum	IVA 2 cy + Surgery + 4 cy of Adriamycin	6	Imatinib	Alive	72
Shen *et al.[40]*	2013	60	F	14.0	Liver	Uncertain malignant potential	Meta M+	Surgery	108	Lung; Liver; Bladder; Pancreas	Surgery	-	-	Alive	120
Dickson *et al.[96]*	2013	24	F	25.0	Retroperitoneum	-	LA	Sirolimus	NA	NA	NA	CR	NA	Alive	22

Dickson *et al.*[96]	2013	40	F	6.0	Retroperitoneum	-	Synch M+	NA	NA	NA	NA	CR	NA	Alive	16
Dickson *et al.*[96]	2013	57	M	10.5	GI	-	Meta M+	Surgery	10	Peritoneum	-	-	-	Alive	14
Dickson *et al.*[96]	2013	37	F	-	Liver	-	LA	2 mos Everolimus + Surgery	-	Local relapse	Surgery	CR	NA	Alive	6
Dickson *et al.*[96]	2013	65	M	-	Adrenal Gland	-	Synch M+	NA	NA	Lung; Soft tissue	Sirolimus	-	Sorafenib > mTOR I	DOD	36
Scheppach *et al.*[41]	2013	23	M	5.5	GI	Malignant	Synch M+	NA	NA	LN; Liver	Surgery + Sirolimus	4	Surgery + CT (AI)	DOD	23
Wyluda *et al.*[42]	2013	31	F	-	Kidney	Malignant	Meta M+	Surgery	84	Lung; LN; Liver; Pancreas; Uterus	Temsirolimus	-	-	DOD	5
Fu *et al.*[43]	2013	38	F	9.9	GI	Malignant	LA	Surgery + CT (IVA)	NA	NA	NA	CR	NA	Alive	6
Williamson *et al.*[44]	2013	55	F	5.0	Bladder	Malignant	Meta M+	Surgery	10	Peritoneum	CT	-	-	DOD	12
Le *et al.*[45]	2014	54	F	18.0	Retroperitoneum	-	Synch M+	NA	NA	Lung	Wedge of 3 pulmonary mets	25	CT (Anthra) + RT > Sirolimus	Alive	76
Shi *et al.*[46]	2014	48	M	14.0	Kidney	Malignant	Synch M+	NA	NA	Lung	Surgery	CR	NA	Alive	148
Cossu *et al.*[47]	2014	52	F	-	Uterus	Malignant	Meta M+	Surgery	36	Lung; Liver	BSC	NA	NA	DOD	60
Ghosh *et al.*[48]	2014	57	F	-	Uterus	Malignant	Meta M+	Surgery	84	LN; Pleural; Pelvis	Temsirolimus > Sirolimus	8	BSC	DOD	10
Bergamo *et al.*[49]	2014	31	F	-	Liver	Malignant	LA	Everolimus 6 mos + Surgery	NA	NA	NA	CR	NA	Alive	12

Kapur *et al.[50]*	2014	49	F	13.5	GI	Malignant	Synch M+	NA	NA	Liver; Peritoneum	Palliative surgery + Sirolimus	-	-	Alive	-
Palleschi *et al.[51]*	2014	65	M	2.5	Bladder	Malignant	Synch M+	NA	NA	Bones	Surgery of primary mass + CT (Gem)	-	-	Alive	7
Sbrollini *et al.[52]*	2014	53	M	2.4	Prostate	Malignant	Synch M+	NA	NA	Lung	Surgery	4	Gemcitabine	Alive	6
Bunch *et al.[53]*	2014	19	F	8.0	Uterus	Malignant	LA	Debulking surgery + Temsirolimus + resection remaining mass	NA	NA	NA	CR	NA	Alive	15
Russel *et al.[54]*	2014	27	F	4.2	Bladder	-	Meta M+	Surgery	1	Peritoneum	Surgery	6	Surgery	Alive	-
Lao *et al.[55]*	2015	47	M	5.2	Bones	Malignant	Synch M+	NA	NA	Lung	Palliative RT + CT	-	-	Alive	42
Neofytou *et al.[56]*	2015	24	F	3.0	Kidney	-	Synch M+	NA	NA	Liver	Surgery	CR	NA	Alive	12
Schoolmeester *et al.[57]*	2015	53	F	17.0	Uterus	Malignant	Meta M+	Surgery	2	Peritoneum	Surgery + Cisplatin-based CT	11	Surgery + Sirolimus	Alive	13
Schoolmeester *et al.[57]*	2015	47	F	8.0	Pelvic wall	Malignant	Meta M+	Surgery	15	Other	Surgery	CR	NA	Alive	57
JK Schoolmeester *et al.[57]*	2015	66	F	-	Uterus	Malignant	Meta M+	Surgery	NA	Lung	Surgery	CR	NA	Alive	1
Sun *et al.[58]*	2015	46	F	-	Uterus	-	Meta M+	Surgery	24	Lung; Kidney	Surgery	1	mTOR I	Alive	7
Liang *et al.[59]*	2015	63	M	9.8	Mediastinal	Malignant	Meta M+	Surgery	3	Bones; Mediastinal relapse	CT	-	-	DOD	7
Fitzpatrick *et al.[60]*	2016	61	F	10.7	Uterus	Malignant	Synch M+	NA	NA	Ovary	Surgery	CR	-	Alive	6

Batereau *et al.*[61]	2016	26	F	-	GI	Malignant	Meta M+	Surgery	84	Peritoneum	Surgery	24	Surgery + RT	Alive	168
Batereau *et al.*[61]	2016	49	F	10.0	Soft Tissue	Malignant	LA	Sirolimus	-	NA	NA	CR	NA	Alive	24
Starbuck *et al.*[62]	2016	30	F	26.8	Uterus	-	Meta M+	Surgery + CT (Gem+T)	3	Bones; Pelvic mets	Temsirolimus > Sirolimus	-	-	Alive	36
Starbuck *et al.*[62]	2016	43	F	-	Uterus	-	LA	Surgery + Temsirolimus	-	Local relapse	Surgery	CR	NA	Alive	18
Starbuck *et al.*[62]	2016	64	F	-	Uterus	-	Meta M+	Surgery + CT (Gem+T)	10	Peritoneum	Surgery + Temsirolimus	3	BSC	DOD	17
Gao *et al.*[63]	2016	47	F	-	Uterus	Malignant	Synch M+	NA	NA	Lung; Kidney	Surgery	1	Sirolimus + Sorafenib	Alive	11
Flechter *et al.*[64]	2016	32	F	-	GI	-	Synch M+	NA	NA	Lung; Brain	Everolimus + brain surgery	18	Doxorubicin	DOD	50
Yoo Bee *et al.*[65]	2016	48	F	-	Ovary	-	Synch M+	NA	NA	Lung	Surgery	-	-	Alive	1
Karpathiou *et al.*[66]	2017	25	F	8.0	Bones	Uncertain malignant potential	Meta M+	Surgery + Temsirolimus	72	Lung; LN; Retroperitoneum; Local relapse	CT (Anthra)	6	-	Alive	96
Okamoto *et al.*[67]	2017	44	F	6.9	Uterus	-	Meta M+	Surgery	36	Lung	Surgery	-	-	Alive	60
Machado *et al.*[68]	2017	33	F	10.0	GI	Malignant	Synch M+	NA	NA	Liver; Peritoneum	Emergency surgery + CT (Anthra)	3	HD-I > mTOR I	DOD	30
Kwon *et al.*[69]	2017	62	F	5.5	Uterus	Malignant	Synch M+	NA	NA	Lung; Lower vagina; Bones	Everolimus	-	-	Alive	18
Kwon *et al.*[69]	2017	38	F	4.6	Uterus	-	Synch M+	NA	NA	LN	Surgery	CR	NA	Alive	8
Varan *et al.*[70]	2017	7	M	3.4	Orbital	Malignant	LA relapse	Sirolimus	0.8	Local relapse	IVA	-	-	Alive	12
Raimondi *et al.*[71]	2018	61	M	-	Kidney	Malignant	Meta M+	Surgery	84	Lung	Sirolimus	-	-	Alive	128

Lin *et al.[72]*	2018	28	M	8.9	GI	Malignant	Synch M+	NA	NA	LN	Surgery	49	Surgery	Alive	120
Hul'ova *et al.[73]*	2018	28	F	15.0	Kidney	Malignant	Meta M+	Surgery	15	Peritoneum	Sunitinib (misdiagnosed as chromophobic renal carcinoma)	6	Everolimus	DOD	104
Tynski *et al.[74]*	2018	20	F	7.2	Soft Tissue	Malignant	Synch M+	NA	NA	Brain; Bones	Surgery + Temsirolimus	-	CT	Dead (Sepsis)	32
Lattanzi *et al.[75]*	2018	38	M	6.0	Kidney	Malignant	Meta M+	Surgery	36	Local relapse; Renal fossa	Surgery	6	Surgery > mTOR I	Alive	70
Alnajar *et al.[76]*	2018	44	M	8.3	Soft Tissue	Malignant	Synch M+	NA	NA	Lung; Bones	Knee amputation + palliative RT + Pazopanib	-	-	Alive	12
Shan *et al.[77]*	2018	27	F	6.2	Uterus	-	Meta M+	Surgery	4	LN	Surgery + CT (ICE)	10	-	DOD	14
AlAzab *et al.[78]*	2019	39	M	7.0	Kidney	Malignant	Synch M+	NA	NA	Lung; LN	Surgery + Sunitinib	9	Everolimus > Sunitinib	DOD	24
Szumera-Cieaekiewicz *et al.[79]*	2019	61	F	10.0	Retroperitoneum	Malignant	Synch M+	NA	NA	LN; Peritoneum	Palliative surgery + Sirolimus	-	-	-	2
Gondran *et al.[80]*	2019	17	M	5.5	GI	Uncertain malignant potential	LA	Sirolimus	NA	Local relapse	-	-	-	Alive	42
Liu *et al.[81]*	2019	51	F	8.3	Vagina	Malignant	LA	Surgery + RT + mTOR I	NA	NA	NA	CR	NA	Alive	7
Liu *et al.[81]*	2019	80	F	10.0	Uterus	-	LA relapse	Surgery	1	Local relapse	Surgery + RT + mTOR I	CR	NA	Alive	48

Liu *et al.*[81]	2019	71	F	14.0	Broad ligament	-	Synch M+	NA	NA	Peritoneum	Surgery	4	mTOR I	DOD	12
Yang *et al.*[82]	2020	55	M	20.5	Retroperitoneum	Malignant	Meta M+	Surgery	10	Kidney	Surgery + CT (T+ Bevacizumab)	12	-	DOD	48
Gupta *et al.*[83]	2020	65	F	6.0	Vagina	Malignant	Synch M+	NA	NA	LN; Liver	RT-CT	-	-	-	-
Xu *et al.*[84]	2020	31	F	25.0	Soft Tissue	Malignant	Synch M+	NA	NA	LN; Liver; Adrenal gland	Surgery + Everolimus	3	Apatinib > Everolimus	DOD	18
Torres Luna *et al.*[85]	2020	32	M	20.0	Kidney	Malignant	Synch M+	NA	NA	Liver	Hepatectomy	CR	NA	-	3
Wu *et al.*[99]	2020	37	F	6.0	Bladder	Malignant	Meta M+	Surgery	13	-	-	-	-	Alive	40
Wu *et al.*[99]	2020	31	F	5.5	Bladder	Malignant	Meta M+	Surgery	10	-	-	20	-	DOD	30
Jia *et al.*[100]	2020	35	F	7.0	Retroperitoneum	Malignant	Meta M+	Surgery + Ivermos	9	Local relapse	-	-	-	DOD	18
Jia *et al.*[100]	2020	25	F	25.0	Retroperitoneum	-	LA relapse	Surgery	-	Local relapse	Surgery	CR	NA	Alive	20
Jia *et al.*[100]	2020	35	F	8.0	Pelvic wall	-	Synch M+	NA	NA	-	Palliative RT + CT	-	Ivermos > mTOR I	DOD	33
Jia *et al.*[100]	2020	35	F	8.0	Kidney	Malignant	Meta M+	Surgery	-	Local relapse	Surgery	-	Surgery	DOD	126
Jia *et al.*[100]	2020	45	M	14.0	Kidney	-	LA relapse	Surgery	-	Local relapse	Surgery	CR	NA	Alive	29
Jia *et al.*[100]	2020	25	M	16.7	Kidney	-	Meta M+	Surgery	-	Lung	Surgery	CR	NA	Alive	45
Jia *et al.*[100]	2020	35	F	14.0	Liver	Malignant	Synch M+	NA	NA	Liver	Surgery	-	Surgery + Sirolimus	Alive	151
Jia *et al.*[100]	2020	35	F	13.0	Pelvic wall	-	LA relapse	Surgery	-	Local relapse	Sirolimus	-	-	Alive	64
Uhlenhopp *et al.*[86]	2020	64	F	15.2	GI	Malignant	Synch M+	NA	NA	Liver	Surgery + Sirolimus	8	Pazopanib > mTOR I	DOD	22

Fabbroni *et al.*[87]	2020	61	F	11.0	Retroperitoneum	Malignant	Meta M+	Surgery	24	Lung	Gemcitabine	3	mTOR I	Alive	108
Liapi *et al.*[88]	2021	53	F	6.0	Uterus	Malignant	Synch M+	NA	NA	Bones	Surgery + stereotaxic RT	3	Everolimus > Pazopanib	Alive	39
Zhong *et al.*[89]	2021	33	F	11.8	Bones	Malignant	Synch M+	NA	NA	Bones	Neoadjuvant CT (AI) + surgery of primary tumor	5	Apatinib + Camrelizumab	Alive	23
Gu *et al.*[103]	2021	-	F	-	Uterus	-	LA relapse	Surgery	48	Local relapse	Surgery + CT (ICE)	4	BSC	Alive	55
Gu *et al.*[103]	2021	-	F	-	Uterus	-	Meta M+	Surgery	12	-	Surgery + CT (ICE)	3	BSC	DOD	16
Kopparthy *et al.*[90]	2021	58	F	7.0	Uterus	-	Meta M+	Surgery	6	Lung	Everolimus	2	Brain RT + Nab-sirolimus	Alive	11

Abbreviations: Anthra : Anthracyclines; AI: Anthracycline and Ifosfamide; BSC: Best supportive care; CR: Complete response without any relapse report; CT : Chemotherapy; cy = cycles; DOD: Death of disease; F: Female; fr: fractions; Gem : Gemcitabine; GI: Gastrointestinal; HD-I : High Dose Ifosfamide; ICE: Ifosfamide, Platin (Cisplatin or Carboplatin) and Anthracycline; IVA: Ifosfamide, Vincristine and Anthracycline; LA: Locally Advanced; LN: Lymph Nodes; M: Male; Meta M+ : Metachronous metastasis; mos : months; mTOR I: mTOR inhibitor; NA: Not applicable; RT: Radiotherapy; RT-CT : concomitant radiotherapy and chemotherapy; Synch M+ : Synchronous metastasis; T: Taxane; yrs: years; - : missing data; > : follow by.
